# *catena*-Poly[[(tetra­aqua­cadmium)-μ-4,4′-bipyridine-κ^2^ *N*:*N*′] 4-hy­droxy-3-sulfonato­benzoate monohydrate]

**DOI:** 10.1107/S1600536812018727

**Published:** 2012-05-02

**Authors:** Shan Gao, Seik Weng Ng

**Affiliations:** a Key Laboratory of Functional Inorganic Material Chemistry, Ministry of Education, Heilongjiang University, Harbin 150080, People’s Republic of China; b Department of Chemistry, University of Malaya, 50603 Kuala Lumpur, Malaysia; c Chemistry Department, Faculty of Science, King Abdulaziz University, PO Box 80203 Jeddah, Saudi Arabia

## Abstract

The two independent Cd^II^ atoms in the polymeric title compound, [Cd(C_10_H_8_N_2_)(H_2_O)_4_](C_7_H_4_O_6_S)·H_2_O, lie on twofold rotation axes, and each is coordinated by four water mol­ecules and the N atoms of two 4,4′-bipyridine mol­ecules in an octa­hedral geometry. Bridging gives rise to chains along [101] and [-101]. The 4-hy­droxy-3-sulfonato­benzoate dianions are not connected to the Cd^II^ atoms, but form hydrogen bonds to the coordinated water mol­ecules as well as the lattice water mol­ecule, generating a three-dimensional network.

## Related literature

For the 1,10-phenanthroline-chelated Mn^II^ derivative of 4-hy­droxy-3-sulfonato­benzoic acid, see: Fang *et al.* (2011 ▶).
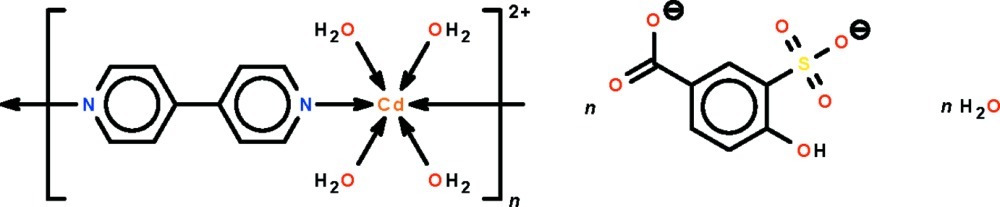


## Experimental

### 

#### Crystal data

[Cd(C_10_H_8_N_2_)(H_2_O)_4_](C_7_H_4_O_6_S)·H_2_O
*M* *_r_* = 574.83
Orthorhombic, 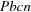

*a* = 16.3246 (10) Å
*b* = 15.3063 (11) Å
*c* = 16.5084 (10) Å
*V* = 4124.9 (5) Å^3^
*Z* = 8
Mo *K*α radiation
μ = 1.23 mm^−1^
*T* = 293 K
0.18 × 0.16 × 0.12 mm

#### Data collection

Rigaku R-AXIS RAPID IP diffractometer
Absorption correction: multi-scan (*ABSCOR*; Higashi, 1995 ▶) *T*_min_ = 0.810, *T*_max_ = 0.867
61947 measured reflections
4705 independent reflections
3503 reflections with *I* > 2σ(*I*)
*R*_int_ = 0.057

#### Refinement

*R*[*F* ^2^ > 2σ(*F* ^2^)] = 0.033
*wR*(*F* ^2^) = 0.090
*S* = 1.04
4705 reflections
334 parameters
11 restraints
H atoms treated by a mixture of independent and constrained refinement
Δρ_max_ = 0.84 e Å^−3^
Δρ_min_ = −0.87 e Å^−3^

### 

Data collection: *RAPID-AUTO* (Rigaku, 1998 ▶); cell refinement: *RAPID-AUTO*; data reduction: *CrystalClear* (Rigaku/MSC, 2002 ▶); program(s) used to solve structure: *SHELXS97* (Sheldrick, 2008 ▶); program(s) used to refine structure: *SHELXL97* (Sheldrick, 2008 ▶); molecular graphics: *X-SEED* (Barbour, 2001 ▶); software used to prepare material for publication: *publCIF* (Westrip, 2010 ▶).

## Supplementary Material

Crystal structure: contains datablock(s) global, I. DOI: 10.1107/S1600536812018727/bt5895sup1.cif

Structure factors: contains datablock(s) I. DOI: 10.1107/S1600536812018727/bt5895Isup2.hkl

Additional supplementary materials:  crystallographic information; 3D view; checkCIF report

## Figures and Tables

**Table 1:** Hydrogen-bond geometry (Å, °)

*D*—H⋯*A*	*D*—H	H⋯*A*	*D*⋯*A*	*D*—H⋯*A*
O1w—H11⋯O5^i^	0.84 (1)	1.93 (1)	2.762 (4)	171 (5)
O1w—H12⋯O1	0.84 (1)	1.99 (2)	2.805 (3)	163 (5)
O2w—H21⋯O6^ii^	0.84 (1)	2.36 (2)	3.136 (3)	154 (4)
O2w—H22⋯O5w^ii^	0.84 (1)	1.85 (1)	2.685 (4)	173 (5)
O3w—H31⋯O2^iii^	0.84 (1)	2.08 (2)	2.858 (3)	155 (3)
O3w—H32⋯O6^iv^	0.84 (1)	1.99 (1)	2.812 (3)	167 (4)
O4w—H41⋯O1^v^	0.84 (1)	1.84 (1)	2.675 (3)	178 (4)
O4w—H42⋯O4^vi^	0.84 (1)	2.10 (2)	2.869 (3)	154 (4)
O5w—H51⋯O2	0.84 (1)	1.96 (2)	2.779 (3)	166 (5)
O5w—H52⋯O6^vii^	0.84 (1)	2.06 (2)	2.850 (4)	157 (6)
O3—H3⋯O2^viii^	0.84 (1)	1.91 (1)	2.746 (3)	176 (4)

Symmetry codes: (i) 
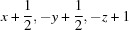
; (ii) 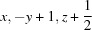; (iii) 
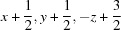
; (iv) 
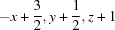
; (v) 
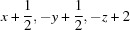
; (vi) 
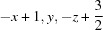
; (vii) 
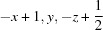
; (viii) 
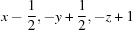
.

**Fig. 1.**
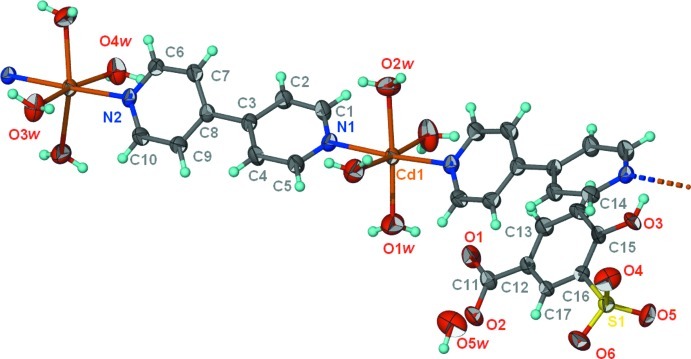
Anisotropic displacement ellipsoid plot (Barbour, 2001) of a portion of the structure of polymeric [Cd(H2O)4(C10H8N2)]2+.(C7H4O6S)2–.H2O at the 70% probability level; hydrogen atoms are drawn as spheres of arbitrary radius.

## supplementary crystallographic information

### Comment

The doubly-deprotonated 4-hydroxy-3-sulfonatobenzoic acid ion functions in a chelating mode to connect Mn^II^ atoms into a chain motif. In this coordination polymer, the metal atom is chelated by 1,10-phenanthroline. When the *N*-heterocycle is replaced by 4,4'-bipyridine in the present synthesis (and with Cd replacing Mn), the dianion is now connected only indirectly, in an outer-sphere type of coordination. The two independent Cd atoms in polymeric [Cd(H_2_O)_4_(C_10_H_8_N_2_)]^2+.^(C_7_H_4_O_6_S)^2–.^H_2_O lie on twofold rotation axes, and each is coordinated by four water molecules and the N atoms of two 4,4'-bipyridine molecules in an octahedral geometry (Fig. 1). *µ*-Bridging gives rise to a chain along [-1 0 1]. The C_7_H_4_O_6_S^2–^ dianion interacts indirectly through the coordinated water molecules as well as through the lattice water molecule to generate a three-dimensional network (Table 1).

### Experimental

A methanol solution (5 ml) of 4,4'-bipyridine (1 mmol) was added to an aqueous solution (10 ml) of cadium(II) dichloride (1 mmol), 2-hydroxy-5-carboxybenzenesulfonic acid (2 mmol) and lithium hydroxide (4 mmol). Colorless crystals were isolated from the solution after several days.

### Refinement

Carbon bound H-atoms were placed in calculated positions (C–H 0.93 Å) and were included in the refinement in the riding model approximation, with *U*(H) set to 1.2*U*(C). The water and hydroxy H-atoms were located in a difference Fourier map, and were refined isotropically with a distance restraint of O–H 0.84±0.01 Å.

Omitted from the refinement is the (20 2 1) reflection.

### Crystal data

**Table:**

[Cd(C_10_H_8_N_2_)(H_2_O)_4_](C_7_H_4_O_6_S)·H_2_O	*F*(000) = 2320
*M**_r_* = 574.83	*D*_x_ = 1.851 Mg m^−^^3^
Orthorhombic, *P**b**c**n*	Mo *K*α radiation, λ = 0.71073 Å
Hall symbol: -P 2n 2ab	Cell parameters from 29441 reflections
*a* = 16.3246 (10) Å	θ = 3.1–27.5°
*b* = 15.3063 (11) Å	µ = 1.23 mm^−^^1^
*c* = 16.5084 (10) Å	*T* = 293 K
*V* = 4124.9 (5) Å^3^	Prism, colorless
*Z* = 8	0.18 × 0.16 × 0.12 mm

### Data collection

**Table:**

Rigaku R-AXIS RAPID IP diffractometer	4705 independent reflections
Radiation source: fine-focus sealed tube	3503 reflections with *I* > 2σ(*I*)
Graphite monochromator	*R*_int_ = 0.057
ω scan	θ_max_ = 27.5°, θ_min_ = 3.1°
Absorption correction: multi-scan (*ABSCOR*; Higashi, 1995)	*h* = −21→21
*T*_min_ = 0.810, *T*_max_ = 0.867	*k* = −19→19
61947 measured reflections	*l* = −21→21

### Refinement

**Table:**

Refinement on *F*^2^	Primary atom site location: structure-invariant direct methods
Least-squares matrix: full	Secondary atom site location: difference Fourier map
*R*[*F*^2^ > 2σ(*F*^2^)] = 0.033	Hydrogen site location: inferred from neighbouring sites
*wR*(*F*^2^) = 0.090	H atoms treated by a mixture of independent and constrained refinement
*S* = 1.04	*w* = 1/[σ^2^(*F*_o_^2^) + (0.0521*P*)^2^ + 1.0723*P*] where *P* = (*F*_o_^2^ + 2*F*_c_^2^)/3
4705 reflections	(Δ/σ)_max_ = 0.001
334 parameters	Δρ_max_ = 0.84 e Å^−^^3^
11 restraints	Δρ_min_ = −0.87 e Å^−^^3^

### Fractional atomic coordinates and isotropic or equivalent isotropic displacement parameters (Å^2^)

**Table:**

	*x*	*y*	*z*	*U*_iso_*/*U*_eq_	
Cd1	0.5000	0.440537 (18)	0.7500	0.02271 (9)	
Cd2	1.0000	0.521170 (18)	1.2500	0.02318 (9)	
S1	0.27334 (4)	0.28183 (4)	0.28084 (4)	0.02442 (16)	
O1	0.47567 (13)	0.21091 (14)	0.60407 (12)	0.0361 (5)	
O2	0.52506 (13)	0.20519 (13)	0.47897 (12)	0.0312 (5)	
O3	0.15128 (12)	0.29724 (14)	0.41107 (13)	0.0340 (5)	
H3	0.1118 (15)	0.299 (2)	0.4435 (18)	0.058 (12)*	
O4	0.23985 (15)	0.36795 (13)	0.26880 (13)	0.0434 (6)	
O5	0.21871 (16)	0.21217 (13)	0.25558 (11)	0.0375 (6)	
O6	0.35463 (14)	0.27325 (16)	0.24423 (11)	0.0402 (6)	
O1W	0.57110 (17)	0.34037 (17)	0.67867 (17)	0.0513 (6)	
H11	0.613 (2)	0.322 (3)	0.702 (3)	0.108 (19)*	
H12	0.544 (3)	0.296 (2)	0.665 (3)	0.088 (17)*	
O2W	0.43176 (16)	0.55653 (16)	0.81748 (14)	0.0393 (5)	
H21	0.398 (2)	0.590 (2)	0.794 (3)	0.087 (16)*	
H22	0.469 (2)	0.589 (3)	0.835 (3)	0.081 (16)*	
O3W	1.06793 (15)	0.62827 (16)	1.17301 (14)	0.0417 (5)	
H31	1.0408 (19)	0.650 (2)	1.1351 (15)	0.047 (11)*	
H32	1.090 (2)	0.668 (2)	1.201 (2)	0.075 (14)*	
O4W	0.91413 (15)	0.41573 (15)	1.30228 (15)	0.0397 (5)	
H41	0.934 (2)	0.376 (2)	1.331 (2)	0.075 (14)*	
H42	0.880 (2)	0.396 (3)	1.270 (2)	0.067 (14)*	
O5W	0.55474 (18)	0.35179 (16)	0.38410 (18)	0.0548 (7)	
H51	0.549 (3)	0.313 (2)	0.420 (2)	0.088 (16)*	
H52	0.592 (2)	0.338 (4)	0.351 (3)	0.12 (2)*	
N1	0.59742 (14)	0.45225 (15)	0.85125 (13)	0.0250 (5)	
N2	0.90277 (14)	0.52540 (14)	1.14842 (13)	0.0233 (5)	
C1	0.58037 (17)	0.44761 (19)	0.93036 (17)	0.0295 (6)	
H1	0.5280	0.4308	0.9461	0.035*	
C2	0.63744 (18)	0.46683 (19)	0.98969 (17)	0.0283 (6)	
H2	0.6228	0.4640	1.0441	0.034*	
C3	0.71664 (17)	0.49032 (17)	0.96802 (16)	0.0237 (6)	
C4	0.73372 (18)	0.4948 (2)	0.88558 (17)	0.0318 (6)	
H4	0.7857	0.5106	0.8679	0.038*	
C5	0.67325 (18)	0.47571 (19)	0.83037 (17)	0.0313 (7)	
H5	0.6859	0.4794	0.7755	0.038*	
C6	0.82516 (17)	0.54887 (18)	1.16227 (16)	0.0259 (6)	
H6	0.8120	0.5719	1.2127	0.031*	
C7	0.76329 (17)	0.54064 (17)	1.10549 (17)	0.0270 (6)	
H7	0.7102	0.5577	1.1182	0.032*	
C8	0.78081 (16)	0.50689 (17)	1.02957 (16)	0.0228 (5)	
C9	0.86225 (18)	0.48489 (19)	1.01436 (17)	0.0297 (6)	
H9	0.8775	0.4641	0.9636	0.036*	
C10	0.92024 (17)	0.4940 (2)	1.07458 (17)	0.0307 (6)	
H10	0.9739	0.4777	1.0634	0.037*	
C11	0.46607 (17)	0.21484 (17)	0.52932 (17)	0.0257 (6)	
C12	0.38208 (16)	0.23377 (16)	0.49698 (15)	0.0230 (5)	
C13	0.31673 (17)	0.24312 (19)	0.55095 (16)	0.0291 (6)	
H13	0.3255	0.2352	0.6061	0.035*	
C14	0.23932 (17)	0.26394 (19)	0.52330 (18)	0.0314 (6)	
H14	0.1963	0.2694	0.5599	0.038*	
C15	0.22524 (16)	0.27683 (17)	0.44052 (16)	0.0247 (6)	
C16	0.29036 (16)	0.26767 (17)	0.38635 (15)	0.0219 (5)	
C17	0.36795 (15)	0.24627 (17)	0.41485 (15)	0.0226 (5)	
H17	0.4110	0.2402	0.3784	0.027*	

### Atomic displacement parameters (Å^2^)

**Table:**

	*U*^11^	*U*^22^	*U*^33^	*U*^12^	*U*^13^	*U*^23^
Cd1	0.01869 (15)	0.02822 (17)	0.02123 (15)	0.000	−0.00454 (10)	0.000
Cd2	0.01997 (16)	0.02926 (17)	0.02031 (15)	0.000	−0.00523 (10)	0.000
S1	0.0210 (3)	0.0301 (3)	0.0222 (3)	0.0000 (3)	−0.0007 (3)	−0.0001 (3)
O1	0.0266 (10)	0.0589 (15)	0.0229 (10)	−0.0022 (9)	−0.0043 (9)	0.0086 (9)
O2	0.0193 (9)	0.0450 (12)	0.0293 (10)	0.0032 (9)	0.0011 (8)	0.0042 (9)
O3	0.0146 (10)	0.0531 (14)	0.0344 (11)	0.0061 (9)	0.0003 (9)	−0.0076 (10)
O4	0.0603 (16)	0.0316 (11)	0.0384 (12)	0.0103 (11)	−0.0041 (11)	0.0065 (9)
O5	0.0385 (13)	0.0429 (13)	0.0311 (12)	−0.0131 (10)	−0.0075 (9)	−0.0063 (9)
O6	0.0259 (12)	0.0698 (16)	0.0250 (11)	0.0036 (11)	0.0068 (8)	0.0025 (10)
O1W	0.0437 (16)	0.0475 (15)	0.0626 (17)	0.0170 (13)	−0.0110 (13)	−0.0224 (13)
O2W	0.0416 (15)	0.0423 (13)	0.0341 (12)	0.0117 (12)	0.0008 (11)	−0.0035 (10)
O3W	0.0479 (15)	0.0411 (13)	0.0360 (12)	−0.0127 (11)	−0.0121 (11)	0.0125 (11)
O4W	0.0333 (13)	0.0401 (13)	0.0457 (14)	−0.0076 (10)	−0.0082 (11)	0.0139 (11)
O5W	0.0587 (18)	0.0380 (14)	0.0675 (18)	0.0051 (12)	0.0140 (15)	0.0150 (13)
N1	0.0229 (12)	0.0302 (12)	0.0220 (11)	0.0000 (10)	−0.0062 (10)	−0.0019 (10)
N2	0.0215 (12)	0.0281 (12)	0.0202 (11)	−0.0024 (9)	−0.0063 (9)	0.0011 (9)
C1	0.0218 (14)	0.0377 (16)	0.0290 (15)	−0.0049 (12)	−0.0015 (12)	0.0035 (12)
C2	0.0270 (15)	0.0381 (15)	0.0198 (13)	−0.0031 (12)	−0.0010 (11)	0.0020 (12)
C3	0.0226 (14)	0.0263 (13)	0.0222 (12)	0.0002 (11)	−0.0046 (12)	0.0001 (10)
C4	0.0192 (14)	0.0518 (17)	0.0245 (14)	−0.0046 (13)	−0.0006 (11)	0.0010 (13)
C5	0.0239 (15)	0.0512 (19)	0.0189 (13)	−0.0016 (13)	−0.0019 (11)	−0.0010 (12)
C6	0.0257 (15)	0.0301 (14)	0.0219 (13)	−0.0033 (12)	0.0003 (11)	−0.0031 (11)
C7	0.0202 (14)	0.0355 (15)	0.0252 (14)	−0.0009 (12)	−0.0019 (11)	−0.0003 (12)
C8	0.0214 (13)	0.0257 (13)	0.0213 (12)	−0.0005 (11)	−0.0041 (12)	0.0026 (10)
C9	0.0239 (15)	0.0411 (17)	0.0240 (13)	0.0035 (12)	−0.0031 (12)	−0.0047 (12)
C10	0.0217 (14)	0.0425 (16)	0.0278 (15)	0.0033 (13)	−0.0029 (12)	−0.0040 (13)
C11	0.0206 (14)	0.0292 (14)	0.0273 (14)	−0.0024 (11)	0.0014 (12)	0.0059 (11)
C12	0.0179 (13)	0.0266 (13)	0.0246 (13)	−0.0010 (11)	−0.0008 (10)	0.0017 (11)
C13	0.0260 (15)	0.0388 (16)	0.0225 (13)	−0.0004 (13)	0.0001 (11)	0.0026 (12)
C14	0.0248 (15)	0.0430 (17)	0.0263 (14)	0.0013 (13)	0.0089 (12)	−0.0015 (12)
C15	0.0155 (12)	0.0287 (14)	0.0298 (14)	0.0007 (11)	−0.0031 (11)	−0.0027 (11)
C16	0.0213 (13)	0.0229 (13)	0.0217 (12)	−0.0014 (10)	0.0001 (11)	−0.0020 (10)
C17	0.0172 (13)	0.0238 (13)	0.0268 (13)	−0.0016 (10)	0.0028 (11)	−0.0035 (11)

### Geometric parameters (Å, º)

**Table:**

Cd1—O1W^i^	2.255 (2)	N1—C1	1.337 (3)
Cd1—O1W	2.255 (2)	N2—C6	1.337 (3)
Cd1—N1	2.314 (2)	N2—C10	1.341 (3)
Cd1—N1^i^	2.314 (2)	C1—C2	1.383 (4)
Cd1—O2W	2.374 (2)	C1—H1	0.9300
Cd1—O2W^i^	2.374 (2)	C2—C3	1.389 (4)
Cd2—O4W	2.305 (2)	C2—H2	0.9300
Cd2—O4W^ii^	2.305 (2)	C3—C4	1.391 (4)
Cd2—N2^ii^	2.310 (2)	C3—C8	1.481 (4)
Cd2—N2	2.310 (2)	C4—C5	1.375 (4)
Cd2—O3W^ii^	2.352 (2)	C4—H4	0.9300
Cd2—O3W	2.352 (2)	C5—H5	0.9300
S1—O4	1.441 (2)	C6—C7	1.384 (4)
S1—O5	1.451 (2)	C6—H6	0.9300
S1—O6	1.464 (2)	C7—C8	1.385 (4)
S1—C16	1.777 (3)	C7—H7	0.9300
O1—C11	1.245 (3)	C8—C9	1.394 (4)
O2—C11	1.281 (4)	C9—C10	1.380 (4)
O3—C15	1.338 (3)	C9—H9	0.9300
O3—H3	0.838 (10)	C10—H10	0.9300
O1W—H11	0.838 (10)	C11—C12	1.500 (4)
O1W—H12	0.842 (10)	C12—C17	1.388 (3)
O2W—H21	0.842 (10)	C12—C13	1.397 (4)
O2W—H22	0.840 (10)	C13—C14	1.381 (4)
O3W—H31	0.835 (10)	C13—H13	0.9300
O3W—H32	0.840 (10)	C14—C15	1.400 (4)
O4W—H41	0.838 (10)	C14—H14	0.9300
O4W—H42	0.835 (10)	C15—C16	1.396 (4)
O5W—H51	0.842 (10)	C16—C17	1.390 (3)
O5W—H52	0.840 (10)	C17—H17	0.9300
N1—C5	1.334 (4)		
			
O1W^i^—Cd1—O1W	94.32 (16)	C6—N2—Cd2	122.33 (17)
O1W^i^—Cd1—N1	91.68 (9)	C10—N2—Cd2	120.25 (18)
O1W—Cd1—N1	94.36 (9)	N1—C1—C2	122.7 (3)
O1W^i^—Cd1—N1^i^	94.36 (9)	N1—C1—H1	118.7
O1W—Cd1—N1^i^	91.68 (9)	C2—C1—H1	118.7
N1—Cd1—N1^i^	171.11 (11)	C1—C2—C3	120.0 (3)
O1W^i^—Cd1—O2W	91.26 (10)	C1—C2—H2	120.0
O1W—Cd1—O2W	174.42 (10)	C3—C2—H2	120.0
N1—Cd1—O2W	85.73 (8)	C2—C3—C4	116.8 (3)
N1^i^—Cd1—O2W	87.62 (8)	C2—C3—C8	121.7 (2)
O1W^i^—Cd1—O2W^i^	174.42 (10)	C4—C3—C8	121.4 (3)
O1W—Cd1—O2W^i^	91.26 (10)	C5—C4—C3	119.6 (3)
N1—Cd1—O2W^i^	87.62 (8)	C5—C4—H4	120.2
N1^i^—Cd1—O2W^i^	85.73 (8)	C3—C4—H4	120.2
O2W—Cd1—O2W^i^	83.17 (12)	N1—C5—C4	123.5 (3)
O4W—Cd2—O4W^ii^	91.14 (13)	N1—C5—H5	118.2
O4W—Cd2—N2^ii^	99.54 (8)	C4—C5—H5	118.2
O4W^ii^—Cd2—N2^ii^	82.74 (8)	N2—C6—C7	123.5 (2)
O4W—Cd2—N2	82.74 (8)	N2—C6—H6	118.3
O4W^ii^—Cd2—N2	99.54 (8)	C7—C6—H6	118.3
N2^ii^—Cd2—N2	176.79 (11)	C6—C7—C8	119.7 (3)
O4W—Cd2—O3W^ii^	89.94 (9)	C6—C7—H7	120.1
O4W^ii^—Cd2—O3W^ii^	167.66 (8)	C8—C7—H7	120.1
N2^ii^—Cd2—O3W^ii^	84.96 (8)	C7—C8—C9	116.7 (2)
N2—Cd2—O3W^ii^	92.80 (8)	C7—C8—C3	122.6 (3)
O4W—Cd2—O3W	167.66 (8)	C9—C8—C3	120.6 (3)
O4W^ii^—Cd2—O3W	89.94 (9)	C10—C9—C8	120.0 (3)
N2^ii^—Cd2—O3W	92.80 (8)	C10—C9—H9	120.0
N2—Cd2—O3W	84.96 (8)	C8—C9—H9	120.0
O3W^ii^—Cd2—O3W	91.63 (13)	N2—C10—C9	123.1 (3)
O4—S1—O5	113.53 (15)	N2—C10—H10	118.5
O4—S1—O6	111.66 (14)	C9—C10—H10	118.5
O5—S1—O6	111.87 (14)	O1—C11—O2	122.9 (3)
O4—S1—C16	107.83 (13)	O1—C11—C12	118.5 (2)
O5—S1—C16	106.75 (12)	O2—C11—C12	118.6 (3)
O6—S1—C16	104.60 (12)	C17—C12—C13	118.8 (2)
C15—O3—H3	118 (3)	C17—C12—C11	121.7 (2)
Cd1—O1W—H11	114 (4)	C13—C12—C11	119.4 (2)
Cd1—O1W—H12	115 (4)	C14—C13—C12	120.8 (2)
H11—O1W—H12	106 (5)	C14—C13—H13	119.6
Cd1—O2W—H21	123 (3)	C12—C13—H13	119.6
Cd1—O2W—H22	105 (3)	C13—C14—C15	120.4 (3)
H21—O2W—H22	106 (4)	C13—C14—H14	119.8
Cd2—O3W—H31	116 (3)	C15—C14—H14	119.8
Cd2—O3W—H32	114 (3)	O3—C15—C16	118.5 (2)
H31—O3W—H32	111 (4)	O3—C15—C14	122.4 (3)
Cd2—O4W—H41	119 (3)	C16—C15—C14	119.1 (2)
Cd2—O4W—H42	115 (3)	C17—C16—C15	120.0 (2)
H41—O4W—H42	111 (4)	C17—C16—S1	120.2 (2)
H51—O5W—H52	111 (5)	C15—C16—S1	119.8 (2)
C5—N1—C1	117.4 (2)	C12—C17—C16	120.9 (2)
C5—N1—Cd1	118.16 (18)	C12—C17—H17	119.5
C1—N1—Cd1	123.94 (19)	C16—C17—H17	119.5
C6—N2—C10	117.0 (2)		
			
O1W^i^—Cd1—N1—C5	152.5 (2)	C4—C3—C8—C7	150.8 (3)
O1W—Cd1—N1—C5	58.0 (2)	C2—C3—C8—C9	145.6 (3)
O2W—Cd1—N1—C5	−116.4 (2)	C4—C3—C8—C9	−32.3 (4)
O2W^i^—Cd1—N1—C5	−33.0 (2)	C7—C8—C9—C10	2.4 (4)
O1W^i^—Cd1—N1—C1	−36.2 (2)	C3—C8—C9—C10	−174.7 (3)
O1W—Cd1—N1—C1	−130.6 (2)	C6—N2—C10—C9	−0.4 (4)
O2W—Cd1—N1—C1	55.0 (2)	Cd2—N2—C10—C9	171.9 (2)
O2W^i^—Cd1—N1—C1	138.3 (2)	C8—C9—C10—N2	−1.5 (5)
O4W—Cd2—N2—C6	62.7 (2)	O1—C11—C12—C17	174.6 (2)
O4W^ii^—Cd2—N2—C6	152.6 (2)	O2—C11—C12—C17	−4.0 (4)
O3W^ii^—Cd2—N2—C6	−26.9 (2)	O1—C11—C12—C13	−2.4 (4)
O3W—Cd2—N2—C6	−118.3 (2)	O2—C11—C12—C13	179.0 (2)
O4W—Cd2—N2—C10	−109.3 (2)	C17—C12—C13—C14	0.4 (4)
O4W^ii^—Cd2—N2—C10	−19.3 (2)	C11—C12—C13—C14	177.5 (3)
O3W^ii^—Cd2—N2—C10	161.2 (2)	C12—C13—C14—C15	−0.6 (4)
O3W—Cd2—N2—C10	69.8 (2)	C13—C14—C15—O3	−179.9 (3)
C5—N1—C1—C2	0.7 (4)	C13—C14—C15—C16	0.5 (4)
Cd1—N1—C1—C2	−170.7 (2)	O3—C15—C16—C17	−179.8 (2)
N1—C1—C2—C3	−1.4 (5)	C14—C15—C16—C17	−0.2 (4)
C1—C2—C3—C4	1.2 (4)	O3—C15—C16—S1	−0.8 (3)
C1—C2—C3—C8	−176.7 (3)	C14—C15—C16—S1	178.9 (2)
C2—C3—C4—C5	−0.4 (4)	O4—S1—C16—C17	−123.5 (2)
C8—C3—C4—C5	177.5 (3)	O5—S1—C16—C17	114.2 (2)
C1—N1—C5—C4	0.2 (4)	O6—S1—C16—C17	−4.5 (3)
Cd1—N1—C5—C4	172.1 (2)	O4—S1—C16—C15	57.5 (2)
C3—C4—C5—N1	−0.3 (5)	O5—S1—C16—C15	−64.9 (2)
C10—N2—C6—C7	1.3 (4)	O6—S1—C16—C15	176.4 (2)
Cd2—N2—C6—C7	−170.8 (2)	C13—C12—C17—C16	0.0 (4)
N2—C6—C7—C8	−0.3 (4)	C11—C12—C17—C16	−177.1 (2)
C6—C7—C8—C9	−1.6 (4)	C15—C16—C17—C12	−0.1 (4)
C6—C7—C8—C3	175.5 (2)	S1—C16—C17—C12	−179.1 (2)
C2—C3—C8—C7	−31.4 (4)		

Symmetry codes: (i) −*x*+1, *y*, −*z*+3/2; (ii) −*x*+2, *y*, −*z*+5/2.

### Hydrogen-bond geometry (Å, º)

**Table:**

*D*—H···*A*	*D*—H	H···*A*	*D*···*A*	*D*—H···*A*
O1w—H11···O5^iii^	0.84 (1)	1.93 (1)	2.762 (4)	171 (5)
O1w—H12···O1	0.84 (1)	1.99 (2)	2.805 (3)	163 (5)
O2w—H21···O6^iv^	0.84 (1)	2.36 (2)	3.136 (3)	154 (4)
O2w—H22···O5w^iv^	0.84 (1)	1.85 (1)	2.685 (4)	173 (5)
O3w—H31···O2^v^	0.84 (1)	2.08 (2)	2.858 (3)	155 (3)
O3w—H32···O6^vi^	0.84 (1)	1.99 (1)	2.812 (3)	167 (4)
O4w—H41···O1^vii^	0.84 (1)	1.84 (1)	2.675 (3)	178 (4)
O4w—H42···O4^i^	0.84 (1)	2.10 (2)	2.869 (3)	154 (4)
O5w—H51···O2	0.84 (1)	1.96 (2)	2.779 (3)	166 (5)
O5w—H52···O6^viii^	0.84 (1)	2.06 (2)	2.850 (4)	157 (6)
O3—H3···O2^ix^	0.84 (1)	1.91 (1)	2.746 (3)	176 (4)

Symmetry codes: (i) −*x*+1, *y*, −*z*+3/2; (iii) *x*+1/2, −*y*+1/2, −*z*+1; (iv) *x*, −*y*+1, *z*+1/2; (v) *x*+1/2, *y*+1/2, −*z*+3/2; (vi) −*x*+3/2, *y*+1/2, *z*+1; (vii) *x*+1/2, −*y*+1/2, −*z*+2; (viii) −*x*+1, *y*, −*z*+1/2; (ix) *x*−1/2, −*y*+1/2, −*z*+1.
